# Bioinspired Bare Bones Mayfly Algorithm for Large-Scale Spherical Minimum Spanning Tree

**DOI:** 10.3389/fbioe.2022.830037

**Published:** 2022-03-01

**Authors:** Tian Zhang, Yongquan Zhou, Guo Zhou, Wu Deng, Qifang Luo

**Affiliations:** ^1^ College of Artificial Intelligence, Guangxi University for Nationalities, Nanning, China; ^2^ Guangxi Key Laboratories of Hybrid Computation and IC Design Analysis, Nanning, China; ^3^ Department of Science and Technology Teaching, China University of Political Science and Law, Beijing, China; ^4^ College of Electronic Information and Automation, Civil Aviation University of China, Tianjin, China

**Keywords:** mayfly algorithm, bare bones mayfly algorithm, large-scale spherical MST, Prüfer code, bioinspired algorithm

## Abstract

Mayfly algorithm (MA) is a bioinspired algorithm based on population proposed in recent years and has been applied to many engineering problems successfully. However, it has too many parameters, which makes it difficult to set and adjust a set of appropriate parameters for different problems. In order to avoid adjusting parameters, a bioinspired bare bones mayfly algorithm (BBMA) is proposed. The BBMA adopts Gaussian distribution and Lévy flight, which improves the convergence speed and accuracy of the algorithm and makes better exploration and exploitation of the search region. The minimum spanning tree (MST) problem is a classic combinatorial optimization problem. This study provides a mathematical model for solving a variant of the MST problem, in which all points and solutions are on a sphere. Finally, the BBMA is used to solve the large-scale spherical MST problems. By comparing and analyzing the results of BBMA and other swarm intelligence algorithms in sixteen scales, the experimental results illustrate that the proposed algorithm is superior to other algorithms for the MST problems on a sphere.

## Introduction

Tree is a connected graph with simple structure which contains no loops and widely applied in graph theory (Diestel, 2000). The minimum spanning tree (MST) problem is a practical, well-known, and widely studied problem in the field of combinatorial optimization (Graham and Hell, 1985). This problem has a long history, which was first put forward by Borüvka in 1926. Many engineering problems are solved based on MST (Bo Jiang, 2009), such as communications network design (Hsinghua et al., 2001), the construction of urban roads, the shortest path (Beardwood et al., 1959), distribution network planning, and pavement crack detection. There are some classical algorithms for solving MST, such as the Prim algorithm (Bo Jiang, 2009) and Kruskal algorithm (Joseph, 1956). They all belong to greedy algorithms, and generally, only one minimum spanning tree can be obtained. However, in practical application, it is usually necessary to find a group of minimum or subminimum spanning trees as the basis for scheme evaluation or selection. Therefore, finding an effective algorithm to solve MST problems is still a frontier topic. In recent years, a large number of bioinspired algorithms have been proposed, such as the marine predator algorithm (Faramarzi et al., 2020), chimp optimization algorithm (Khishe and Mosavi, 2020), arithmetic optimization algorithm (Abualigah et al., 2021), bald eagle search algorithm (Alsattar et al., 2020), Harris hawks optimization algorithm (Heidari et al., 2019), squirrel search algorithm (Jain et al., 2018), pathfinder algorithm (Yapici and Cetinkaya, 2019), equilibrium optimizer (Faramarzi et al., 2019). The swarm intelligence algorithm has been widely used in various optimization problems and achieved good results, for example, path planning problems solved by the central force optimization algorithm (Chen et al., 2016), teaching–learning-based optimization algorithm (Majumder et al., 2021), water wave optimization algorithm (Yan et al., 2021), chicken swarm optimization algorithm (Liang et al., 2020), etc. Location problems are solved by the genetic algorithm (Li et al., 2021), particle swarm optimization (Yue et al., 2019), flower pollination algorithm (Singh and Mittal, 2021), etc. Also, the design of a reconfigurable antenna array is solved by the differential evolution algorithm (Li and Yin, 2011a), biogeography-based optimization (Li and Yin, 2011b), etc. In fact, the meta-heuristic algorithm can generate a set of minimum or subminimum spanning trees rather than one minimum spanning tree. The genetic algorithm (Zhou et al., 1996), artificial bee colony algorithm (Singh, 2009), ant colony optimization (Neumann and Witt, 2007), tabu search algorithm (Katagiri et al., 2012), and simulated annealing algorithm have been used for solving the MST problem.

For the MST problem, we usually calculate it in two-dimensional space, but it is of practical significance to study MST in three-dimensional space. For example, sockets are connected with wires in cuboid rooms, and roads on hills and mountains are planned. Also, as we all know, the surface of the Earth where we live is very close to a sphere. In many research fields, atoms, molecules, and proteins are represented as spheres, and foods in life, such as eggs, seeds, onions, and pumpkins, are close to spheres. Some buildings, glass, and plastics are made into spheres. Similar to the traveling salesman problem (TSP), it is also an NP-hard problem. Now scholars have applied the cuckoo search algorithm (Ouyang et al., 2013), glowworm swarm optimization (Chen et al., 2017), and flower pollination algorithm (Zhou et al., 2019) to solve the spherical TSP. Thus, it is of essence crucial to study the MST on a three-dimensional sphere. Bi and Zhou have applied the improved artificial electric field algorithm to the spherical MST problem (Bi et al., 2021). In this article, we will further study the cases of more nodes on the sphere.

The mayfly algorithm (MA) proposed by Konstantinos Zervoudakis and Stelios Tsafarakis (2020) is a population-based intelligent optimization bioinspired algorithm inspired by the flight and mating behavior of adult mayflies. Due to its high calculation accuracy and simple structure, researchers employed it to address problems of numerous disciplines. Guo and Kittisak Jermsittiparsert used improved MA to optimize the component size of high-temperature PEMFC-powered CCHP (Guo et al., 2021). Liu and Jiang proposed a multiobjective MA for a short-term wind speed forecasting system based on optimal sub-model selection (Liu et al., 2021a). Trinav Bhattacharyya and Bitanu Chatterjee combined MA with harmony search algorithm to solve the feature selection problem (Bhattacharyya et al., 2020). Liu and Chai used energy spectrum statistics and improved MA for bearing fault diagnosis (Liu et al., 2021b). Chen and Song proposed the balanced MA to optimize the configuration of electric vehicle charging stations on the distribution system (Chen et al., 2021). MohamedAbd and ElazizaS. Senthilraja used MA to predict the performance of a solar photovoltaic collector and electrolytic hydrogen production system (AbdElaziz et al., 2021). To obtain a group of more perfect minimum spanning trees or subminimum spanning trees on a sphere in finite time, a bare bones mayfly algorithm (BBMA) is proposed to solve spherical MST problems. By simplifying the algorithm parameters and using the statistical update method, the fast convergence and solution accuracy of the proposed algorithm are better than before, and it shows superior ability in solving large-scale problems.

The rest of this article is organized as follows: *Related Work* describes the related work and basic mayfly algorithm. *The Proposed BBMA for Large-Scale Spherical MST* introduces the proposed bare bones mayfly algorithm for spherical MST. Comparison and analysis of results evaluated by BBMA and other algorithms are given in *Experimental Results and Discussion*. This article is concluded in *Conclusion and Future Work*.

## Related Work

### Spherical Minimum Spanning Tree Mathematical Model

**FIGURE 1:**
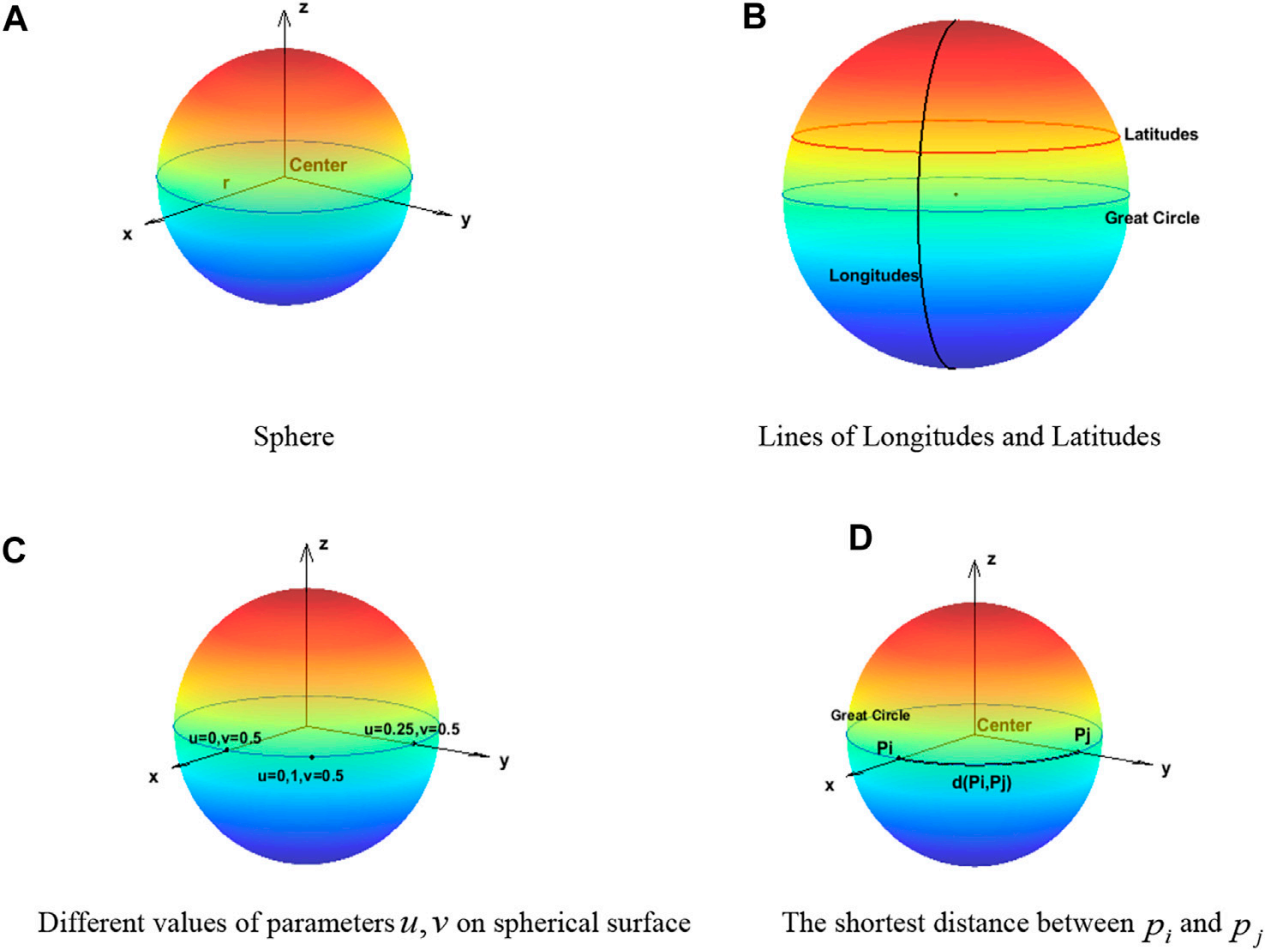
Related definitions on the sphere. **(A)** Sphere. **(B)** Lines of longitudes and latitudes. **(C)** Different values of parameters u,v on the spherical surface. **(D)** The shortest distance between $p_{i}$ and $p_{j}$ .

A semicircle takes its diameter as its axis of rotation, and the surface formed by rotation is called a sphere. The radius of the semicircle is the radius of the sphere. In this study, the coordinate origin (Figures 1A,B ) is set as the center of the sphere. The equation of a sphere with radius r is

$$x^{2}+y^{2}+z^{2}=r^{2},$$ (1)

where (x,y,z) is the coordinate of each point on the sphere.

#### Representation of Points on a Sphere

The coordinate position on the sphere can be expressed by the following formula (Hearn and Pauline Baker, 2004):

p(u,v)=(x(u,v),y(u,v),z(u,v)). (2)

Each coordinate is represented by a function of the surface parameters u and v . Usually, we normalize the three coordinate functions and make u and v in the range of 0–1. Eqs. 3–5 show a sphere with radius r , and the center is at the coordinate origin (Eldem and Ülker, 2017).

x(u,v)=r⁡cos(2πu)sin(πv), (3)

y(u,v)=r⁡sin(2πu)sin(πv), (4)

z(u,v)=r⁡cos(πv), (5)

where parameters u and v determine a position by representing lines of constant longitude and lines of constant latitude, respectively. To simplify calculations, a sphere with r=1 is used in this study. When the parameters u and v take different values, the coordinate position on the sphere is as shown in Figure 1C (Uğur et al., 2009).

#### Geodesics Between Point Pairs on a Unit Sphere

The circle of a sphere cut by the plane passing through the center of the sphere is called a great circle (Wikipedia, 2012). On the sphere, the length of the shortest connecting line between two points is the length of an inferior arc between the two points of the great circle passing through the two points. We call this arc length the geodesic (Lomnitz, 1995).

The geodesic between two points $p_{i}\left(x_{i},y_{i},z_{i}\right)$ and $p_{j}\left(x_{j},y_{j},z_{j}\right)$ on a sphere is shown in Figure 1D. These two points can be represented by two vectors ${\vec{v}}_{i}=\left(x_{i},y_{i},z_{i}\right)$ and ${\vec{v}}_{j}=\left(x_{j},y_{j},z_{j}\right)$ . The scalar product of the two vectors is

$${\vec{v}}_{i}•{\vec{v}}_{j}=\left|{\vec{v}}_{i}\right|\left|{\vec{v}}_{j}\right|\cos\theta ,$$ (6)

where θ is the angle between two vectors. The scalar product is calculated as

$${\vec{v}}_{i}•{\vec{v}}_{j}=x_{i}x_{j}+y_{i}y_{j}+z_{i}z_{j}.$$ (7)

Also, the shortest distance formula is

$${\hat{d}}_{p_{i},p_{j}}=r\theta .$$ (8)

From Eqs 6–8, we get

$${\hat{d}}_{p_{i},p_{j}}=r\arccos\left(\frac{x_{i}x_{j}+y_{i}y_{j}+z_{i}z_{j}}{r^{2}}\right).$$ (9)

The distance from point $p_{i}$ to point $p_{j}$ is the same as the distance from $p_{j}$ to $p_{i}$ . If there are n points on the sphere, an n×n symmetric distance matrix D will be obtained by calculating the distance between each two points. The matrix D is as follows:

$$D=\left[\begin{array}{cccc} {\hat{d}}_{11} & {\hat{d}}_{12} & \cdots & {\hat{d}}_{1n}\\ {\hat{d}}_{21} & {\hat{d}}_{21} & \cdots & {\hat{d}}_{2n}\\ \vdots & \vdots & \ddots & \vdots \\ {\hat{d}}_{n1} & {\hat{d}}_{n2} & \cdots & {\hat{d}}_{nn} \end{array}\right]=\left[\begin{array}{cccc} \infty & {\hat{d}}_{12} & \cdots & {\hat{d}}_{1n}\\ {\hat{d}}_{21} & \infty & \cdots & {\hat{d}}_{2n}\\ \vdots & \vdots & \ddots & \vdots \\ {\hat{d}}_{n1} & {\hat{d}}_{n2} & \cdots & \infty \end{array}\right],$$ (10)

where ${\hat{d}}_{i,j}$ represents the length of the geodesic between point $p_{i}$ and point $p_{j}$ . In particular, ${\hat{d}}_{i,i}=\infty$ means that point $p_{i}$ cannot reach itself.

#### Spherical Minimum Spanning Tree Mathematical Model

For the two-dimensional MST problem, G=(V,E) represents an undirected graph, where $V=\left\{v_{1},v_{2},\cdots ,v_{n}\right\}$ is a finite set of nodes and $E=\left\{e_{ij}|v_{i},v_{j}\in V\right\}$ is a finite set of edges. Each has its corresponding weight $w_{ij}$ . $x_{ij}\left(i,j=1,2,\cdots ,n\right)$ is set as 0 or 1. If $x_{ij}=1$ , $e_{ij}$ is selected; if $x_{ij}=0$ , $e_{ij}$ is not selected. The variable |S| is the number of nodes of the graph contained in the set S . The mathematical model of the minimum spanning tree is as follows:

$$\min f\left(x\right)=\sum_{i-1}^{n-1}\sum_{j=1+1}^{n}w_{ij}x_{ij},$$ (11)

$$s.t.\sum_{i-1}^{n-1}\sum_{j=1+1}^{n}x_{ij}=n-1,$$ (12)

$$\sum_{v_{i}\in S}\sum_{v_{j}\in S,i<j}x_{ij}\leq \left|S\right|-1,\forall S\subset V,\left|S\right|\geq 2,$$ (13)

$$x_{ij}\in \left\{0,1\right\}\;i,j=1,2,\cdots ,n,$$ (14)

where the constraint condition Eq. 12 ensures that the last generated graph is a spanning tree. Also, constraint condition Eq. 13 ensures that it is not a circle in the process of solving the minimum spanning tree problem.

As for a 3*D* spherical minimum spanning tree problem, a finite set of nodes $\text{P}=\left\{p_{1},p_{2},\cdots ,p_{n}\right\}$ are on a sphere. Each node is represented by $p_{i}=\left(x_{i},y_{i},z_{i}\right)$ . $A=\left\{a_{ij}|p_{i},p_{j}\in V\right\}$ is a finite set of geodesics. Each geodesic has its corresponding weight ${\hat{d}}_{ij}$ which is calculated by Eq. 9. An n×n symmetric distance matrix D can be constructed as shown in Eq. 10. $x_{ij}\left(i,j=1,2,\cdots ,n\right)$ is set as 0 or 1. If $x_{ij}=1$ , $a_{ij}$ is selected; if $x_{ij}=0$ , $a_{ij}$ is not selected. The variable |S| is the number of nodes on a sphere. The mathematical model of the spherical MST is as follows:

$$\min f\left(x\right)=\sum_{i-1}^{n-1}\sum_{j=1+1}^{n}{\hat{d}}_{ij}x_{ij}.$$ (15)

Similarly, constraint condition Eqs 12–14 are applied to Eq. 15.

### Mayfly Algorithm

Mayfly algorithm is a new swarm intelligence bioinspired algorithm proposed in 2020. Its inspiration comes from the flying and mating behavior of male and female mayflies in nature. The algorithm can be considered as a modification of particle swarm optimization (PSO) (Kennedy and Eberhart, 1995), genetic algorithm (GA) (Goldberg, 1989), and firefly algorithm (FA) (Yang, 2009). At present, researchers have applied MA to many engineering problems.

Mayflies are insects that live in water when they are young. The feeding ability will be lost, and they only mate and reproduce when they grow up. In order to attract females, most adult male mayflies gather a few meters above the water to perform a nuptial dance. Then, female mayflies fly into these swarms to mate with male mayflies. After mating, the females lay their eggs on the water, and the mated mayflies will die.

In MA, the two idealized rules should be followed. First, after mayflies are born, they are regarded as adults. Second, the mated mayflies which have stronger ability to adapt to the environment can continue to survive. The algorithm works as follows. First, male and female populations are randomly generated. Each mayfly in the search space is regarded as a candidate solution represented by a d -dimensional vector $X=\left(x_{1},x_{2},\cdots ,x_{d}\right)$ for male and $Y=\left(y_{1},y_{2},\cdots ,y_{d}\right)$ for female. Its performance is evaluated according to the objective function f(⋅) shown in Eq. 15. The velocity of each mayfly is expressed by $V=\left(v_{1},v_{2},\cdots ,v_{d}\right)$ . The flying direction of each male mayfly is guided by its best location in history and the global optimal position in the population. Meanwhile, the female mayflies fly to the corresponding male mayflies. The main steps of mayfly algorithm are described as follows.

#### Movement of Male Mayflies

The gathering of male mayflies in a swarm is always a few meters above water for performing the nuptial dance. The position of a male mayfly is updated as follows:

$$x_{i}^{t+1}=x_{i}^{t}+v_{i}^{t+1},$$ (16)

where $x_{i}^{t}$ is the position of mayfly i at time t and $x_{i}^{t+1}$ is the position at time t+1 and $v_{i}^{t+1}$ is the velocity of mayfly i at time t+1 . The velocity is adjusted by its own velocity and individual and social experiences at time t . However, the best male mayfly in the population is not affected by other mayflies, which helps the algorithm escape the local optimal. The velocity of a male mayfly i is calculated as

$$v_{ij}^{t+1}=\{\begin{array}{c} v_{ij}^{t}+\alpha_{1}\cdot e^{-\beta r_{p}^{2}}\left(pbest_{ij}-x_{ij}^{t}\right)+\alpha_{2}\cdot e^{-\beta r_{g}^{2}}\left(gbest_{j}-x_{ij}^{t}\right),f\left(x_{i}^{t}\right)>f\left(gbest\right)\\ v_{ij}^{t}+d\cdot r,f\left(x_{i}^{t}\right)=f\left(gbest\right) \end{array},$$ (17)

$$r_{p}=\sqrt{\sum_{j=1}^{n}{\left(x_{ij}-pbest_{ij}\right)}^{2}},$$ (18)

$$r_{g}=\sqrt{\sum_{j=1}^{n}{\left(x_{ij}-gbest_{j}\right)}^{2}},$$ (19)

where $v_{ij}^{t}$ represents the velocity of male mayfly i at time t in dimension j(j=1,2,⋯,n) , $x_{ij}^{t}$ represents the position of dimension j of mayfly i at time t , $\alpha_{1}$ and $\alpha_{2}$ represent positive attraction constants used to scale the contribution of the cognitive and social component, respectively, and β is a fixed visibility coefficient used to limit a mayfly’s visibility to others. Furthermore, the best individual historical position of mayfly i is represented by $pbest_{i}$ and gbest is the global best position at time step t , while $r_{p}$ is the Cartesian distance between $x_{i}$ and $pbest_{i}$ and $r_{g}$ is the Cartesian distance between $x_{i}$ and gbest . These distances are calculated according to Eq. 18, 19. Finally, d is the nuptial dance coefficient and r∈[−1,1] is a random value.

#### Movement of Female Mayflies

The female mayflies move toward the males for breeding. The position of a female mayfly is updated as follows:

$$y_{i}^{t+1}=y_{i}^{t}+v_{i}^{t+1},$$ (20)

where $y_{i}^{t}$ is the position of female mayfly i at time t and $y_{i}^{t+1}$ is the position at time step t+1 and $v_{i}^{t+1}$ represents the velocity of female mayfly i at time t+1 . Its velocity is affected by its own velocity and the corresponding male mayfly’s position. It means that according to their fitness function, the best female should be attracted by the best male, the second best female by the second best male, and so on. However, the female mayfly which is better than the corresponding male mayfly is not affected by a male, it flies randomly. Consequently, considering minimization problems, their velocities are calculated as

$$v_{ij}^{t+1}=\{\begin{array}{c} v_{ij}^{t}+\alpha_{2}\cdot e^{-\beta r_{mf}^{2}}\left(x_{ij}^{t}-y_{ij}^{t}\right),f\left(y_{i}^{t}\right)>f\left(x_{i}^{t}\right)\\ v_{ij}^{t}+fl\cdot r,f\left(y_{i}^{t}\right)\leq f\left(x_{i}^{t}\right) \end{array},$$ (21)

$$r_{mf}=\sqrt{\sum_{j=1}^{n}{\left(x_{ij}-y_{ij}\right)}^{2}},$$ (22)

where $v_{ij}^{t}$ is a velocity of female mayfly i at time t , $y_{ij}^{t}$ is the position in dimension j at time t , $\alpha_{2}$ is the positive attraction constant, and β represents an unchanged visibility coefficient. $r_{mf}$ represents the distance between $x_{i}$ and $y_{i}$ calculated according to Eq. 22. Finally, fl is the random fly coefficient, and r∈[−1,1] is a random value.

#### Mating of Mayflies

The mating rules are the same as the way females are attracted by males. The best female breeds with the best male, the second best female with the second best male, and so on. The positions of two offspring are generated by the arithmetic weighted sum of the positions of parents as follows:

$$\begin{array}{c} offspring1=L\cdot male+\left(1-L\right)\cdot female\\ offspring2=L\cdot female+\left(1-L\right)\cdot male \end{array},$$ (23)

where male is the male mayfly’s position, female is the female mayfly’s position, and L∈[−1,1] is a random value. Offspring’s initial velocities are set to be zero, which helps the convergence of the algorithm.

After mating, the offspring are mixed with male and female parents. Then, the fitness values are sorted. The mayflies with low adaptability will die, and those with high adaptability will live for the next iteration. Algorithm 1 shows the pseudocode of MA.

Algorithm 1 Mayfly algorithm.

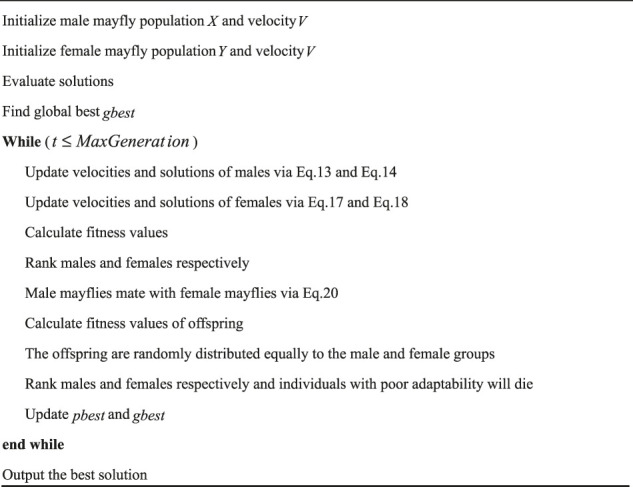

## The Proposed BBMA for Large-Scale Spherical MST

### MST Based on Prüfer Coding

A coding method for marking rootless trees is called Prüfer coding. The initial population generated by this coding method will not produce infeasible solutions after being improved. Prüfer coding is needed to solve the spherical MST. Its idea comes from Cayley’s theorem, which means that there are $n^{n-2}$ different minimum spanning trees for a complete graph with n nodes (Crabb, 2006). It shows that the arrangement of n−2 numbers can uniquely represent a tree, and these numbers are integers between 1 and n . Such an arrangement that can represent a tree is the Prüfer sequence. The process of converting a tree into a Prüfer sequence is as follows:

Step 1: node i is the leaf node with the smallest value on the tree T

Step 2: the node j uniquely connected to i is taken as the first coding number, and the coding order is from left to right
Step 3: node i and the edge from i to j are deleted, and a n−1 node tree is obtained
Step 4: this is repeated until only one edge is left

Through the abovementioned steps, we can get a Prüfer sequence of tree T which is n−2 permutations of the numbers between 1 and n .

### Code Design

We assume that there are n points on a sphere, and these points are represented by different integers between 1 and n . The dimension of the position of each individual is n−2 , and the value in each dimension is a real number between 1 and n .

Suppose an individual is represented by

$$X_{1}:\left(1.75,7.13,3.84,2.12,4.26,5.06\right).$$ (24)

The Prüfer sequence obtained by rounding $X_{1}$ is as follows:

$$X_{1}\to X_{2}:\left(2,7,4,2,4,5\right).$$ (25)

**FIGURE 2:**
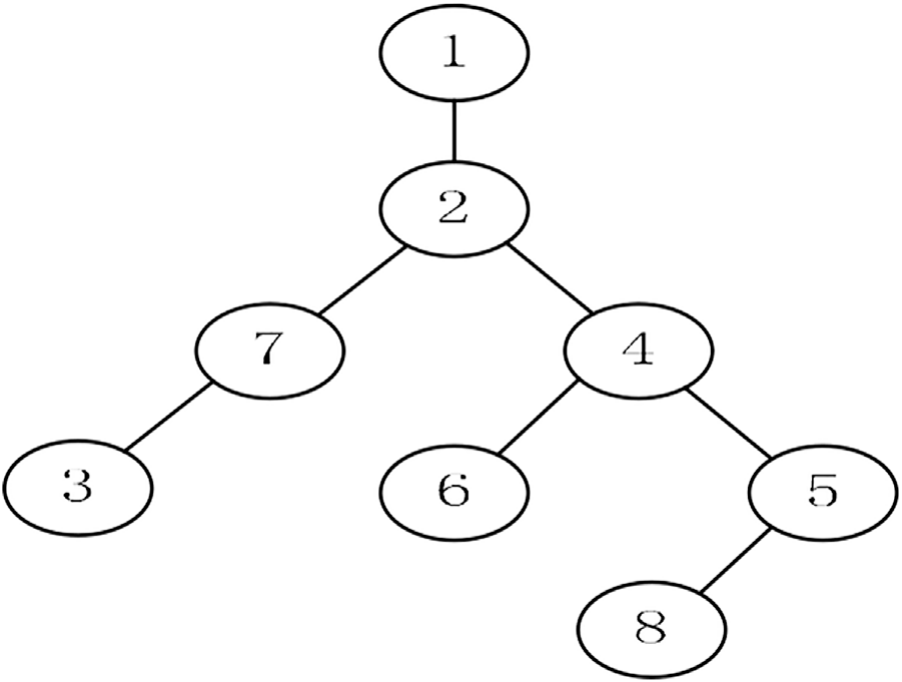
Spanning tree.

According to $X_{2}$ , the spanning tree shown in Figure 2 is obtained.

The pseudo code of decoding the Prüfer sequence into a tree is shown in Algorithm 2.

Algorithm 2 Decoding the Prüfer sequence into a tree.

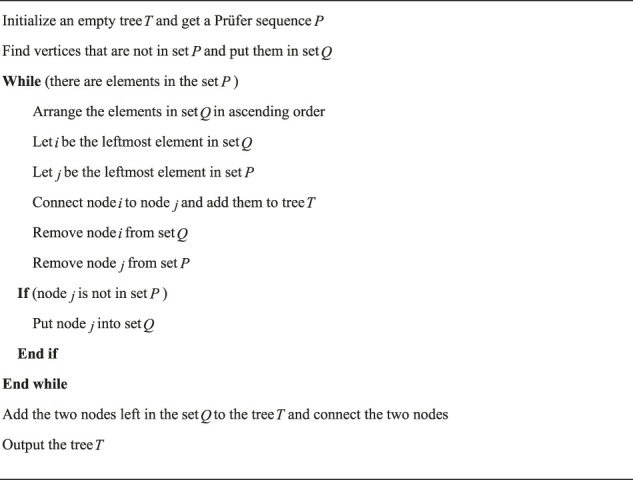

### The BBMA Algorithm

The basic MA has the problem of many initial parameters which have a great impact on the results. Besides, the accuracy of MA is not high enough due to lack of exploitation ability. Bare bones mayfly algorithm avoids the influence of parameters by cancelling the velocity (Ning and Wang, 2020; Song et al., 2020), and individual position is directly obtained by random sampling obeying Gaussian distribution like bare bones PSO (Kennedy, 2003). In order to enhance the exploitation ability and help the algorithm escape from the local optimal solution, BBMA uses Lévy flight to perform the nuptial dance of the optimal male and the random flight of the excellent female (Nezamivand et al., 2018). In addition, individuals crossing the border are pulled back into the search space instead of the method of placing cross-border individuals on the boundary so that it reduces the waste of search space (Wang et al., 2016). The main steps of BBMA are described as follows.

#### Movement of Male Mayflies

Male mayflies can be renewed in two ways as before. First, for individuals who are not the best, the Gaussian distribution based on the global optimal position and individual historical optimal position is used to calculate the position. In order to keep a balance between the diversity and convergence of algorithm, a disturbance which changes adaptively based on the diversity of the population and the convergence degree of the current individual is added (Zhang et al., 2014). The new update strategy is described as follows:

$$x_{i}^{t+1}=N\left(\mu ,\sigma^{2}\right),$$ (26)

$$\mu =\frac{\left(gbest_{j}+pbest_{ij}\right)}{2},\sigma =\left|gbest_{j}-pbest_{ij}\right|+\delta ,$$ (27)

$$\delta =rand\cdot \left|x_{k1,j}-x_{k2,j}\right|\cdot e^{f\left(gbest\right)-f\left(x_{i}\right)},$$ (28)

where $N\left(\mu ,\sigma^{2}\right)$ is the Gaussian distribution with mean μ and standard deviation σ , gbest represents the global optimal individual, $pbest_{i}$ is the historical optimal solution of individual i , rand∈[0,1] is a random value, and $x_{k1}$ and $x_{k2}$ are two solutions selected from other male mayflies at random.

We know that the population is scattered in the early stage of evolution, so σ is large, and the Gaussian distribution is scattered, which is conducive for global search. In the later stage of evolution, the population is relatively concentrated, and individuals search carefully around μ . However, if the pbest of a individual happens to be close or equal to gbest in the evolution process, this individual will stop updating because the variance of Gaussian distribution becomes 0. Also, if most individuals among the swarm stop updating prematurely, the algorithm will converge to a false global optimum with high probability. Thus, assigning a disturbance on the variance of Gaussian distribution is a good way. As shown in Eq. 28, on the assumption that $\left|x_{k1,j}-x_{k2,j}\right|$ remains constant, the smaller the differential fitness value between gbest and $x_{i}$ , the higher the disturbance δ . When the individual has the same fitness as gbest , this individual will be affected by a disturbance with the maximal magnitude. In this case, this disturbance may prevent the algorithm from trapping into a local optimal solution. Furthermore, with the iteration of the algorithm, individuals get denser and denser. The smaller the value of $\left|x_{k1,j}-x_{k2,j}\right|$ , the smaller the δ and σ , which ensures the convergence of the algorithm.

As for the second individual update method, if the individual is the global optimal solution, Lévy flight is adopted. The small step size of Lévy flight improves the exploitation ability of the algorithm, and the less long step increases the ability of avoiding getting stuck in a local optimal value (Dinkar and Deep, 2018; Ren et al., 2021). By using Lévy flight, the overall performance of BBMA in solving large-scale problems has been greatly enhanced. In fact, Lévy flight is a random walk, which follows the Lévy distribution of the following formulas:

$$Levy\left(s\right)∼s^{-1-\beta },0\leq \beta \leq 2,$$ (29)

$$s=\frac{A}{{\left|B\right|}^{1/\beta }},A∼N\left(0,\sigma_{A}^{2}\right),B∼N\left(0,\sigma_{B}^{2}\right),$$ (30)

$$\sigma_{A}={\left(\frac{\Gamma \left(1+\beta \right)\cdot \sin\left(\frac{\pi \beta }{2}\right)}{\Gamma \left(1+\frac{\beta }{2}\right)\cdot \beta \cdot 2^{\frac{\beta -1}{2}}}\right)}^{1/\beta },\sigma_{B}=1,$$ (31)

$$\Gamma \left(1+\beta \right)=\int_{0}^{\infty }t^{\beta }e^{-t}dt,$$ (32)

where s represents the step size and β is an index by which the peak sharpness of the Lévy distribution can be adjusted. In this work, we set β=1.5 . A and B follow the Gaussian distribution, and Γ stands for the gamma function which is obtained by Eq. 32. For the best individual, the update formula is as follows:

$$x_{i}^{t+1}=x_{i}^{t}+x_{i}^{t}\cdot Levy\left(\beta \right).$$ (33)

By using Lévy flight to search the solution space, the global exploration ability and local exploitation ability of the algorithm are better balanced.

#### Movement of Female Mayflies

Female mayflies can be renewed in two ways as before. Firstly, for individuals who are worse than their corresponding male mayflies, the Gaussian distribution based on the current female mayfly’s position and its corresponding male mayfly’s position is used to calculate the position. The new update strategy is described as follows:

$$y_{i}^{t+1}=N\left(\mu ,\sigma^{2}\right),$$ (34)

$$\mu =\frac{\left(x_{ij}+y_{ij}\right)}{2},\sigma =\sqrt{\left|x_{ij}-y_{ij}\right|},$$ (35)

where $N\left(\mu ,\sigma^{2}\right)$ is the Gaussian distribution, $y_{ij}$ is the position of the female mayfly, and $x_{ij}$ is the position of its corresponding male mayfly. The root sign makes the Gaussian distribution relatively concentrated so that it ensures female mayflies approach male mayflies faster, which accelerates the convergence.

As for the second individual update method, if the female mayfly is better than its corresponding male mayfly, the excellent female mayfly, like the best male mayfly, should use the strategy of Lévy flight which will make the algorithm get rid of the local optimum (Barshandeh and Haghzadeh, 2020). For excellent female mayflies, the update formula is as follows:

$$y_{i}^{t+1}=y_{i}^{t}+y_{i}^{t}\cdot Levy\left(\beta \right).$$ (36)

Both Gaussian distribution and Lévy distribution are statistical random distribution. The distribution of the former is regular, and the distribution of the latter is irregular. Their cooperation can prevent the lack of diversity of the algorithm and improve the convergence speed.

#### Mating of Mayflies

The mating process is the same as the basic MA as shown in Eq. 23. After mating, the offspring are mixed with parents. Then, the mayflies with low adaptability will die, and those with high adaptability will live for the next iteration.

#### Handling Cross-Border Mayflies

In the early stage of population evolution, the distance between the historical optimal position and the global optimal position of different individuals is far away, and the standard deviation σ of Gaussian distribution used for updating positions is relatively large, resulting in a greater opportunity for the new position to cross the boundary of the search space. In basic MA, the position of the cross-border individual is directly placed on the boundary, which will result in a waste of resources. In this study, according to the degree of individuals crossing the boundary, with the expectation μ of Gaussian distribution as the center, the cross-border individual x is pulled back to the search space to obtain $x^{\prime }$ , and the cross-border individual is treated according to the following equation:

$$x^{\prime }=\mu +\frac{{\left(x_{border}-\mu \right)}^{2}}{x-\mu },$$ (37)

where $x_{border}$ is the boundary, we assume $x_{\max}$ is the upper bound and $x_{\min}$ is the lower bound, if $x>x_{\max},x_{border}=x_{\max}$ , and if $x<x_{\min},x_{border}=x_{\min}$ . According to Eq. 37, when x crosses the upper bound, it is pulled back to the interval $\left(\mu ,x_{\max}\right)$ . The less the x crosses $x_{\max}$ , the closer it is pulled back to $x_{\max}$ ; the more the x crosses $x_{\max}$ , the closer it is pulled back to the center μ . When x crosses the lower bound, it is pulled back to the interval $\left(x_{\min},\mu \right)$ . Similarly, the degree to which individuals are pulled back into the search space is proportional to the degree of individuals crossing the boundary.

The concrete implementation steps of the bare bones mayfly algorithm for spherical MST are as follows.

Algorithm 3 The BBMA for spherical MST.

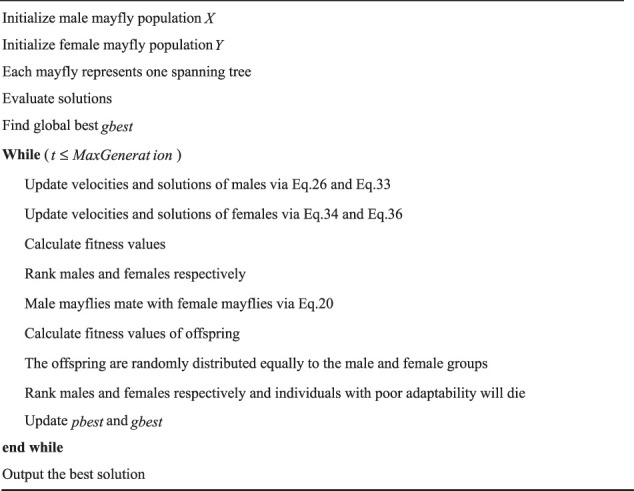

## Experimental Results and Discussion

A large number of cases with different number of points are used to test the ability of BBMA in solving MST problems. All experiments are carried out on a sphere with r=1 , and the number of nodes the sphere is *n* = 25, 50, 75, 100, 150, 200, 250, 300, 350, 400, 500, 600, 700, 800, 900, and 1,000, The data and results of 400 nodes or less come from the literature (Bi et al., 2021) and the node data in higher dimensions are randomly generated. Due to the randomness of meta-heuristic algorithm, each case is run 30 times independently. The structure of this section is as follows: in *Experimental Setup*, the experimental setup is given; *Comparison of Algorithms in Low-Dimensional Cases* shows the comparison and analysis of experimental results between BBMA and other algorithms in the cases of low dimension; the comparison for medium-dimensional cases is shown in *Comparison of Algorithms for Medium-Dimensional Cases*; and the high-dimensional cases are shown in *Comparison of Algorithms for High-Dimensional Cases*.

### Experimental Setup

All of the experiments are compiled in MATLAB R2019a. System specification: an Intel Core i3-6100 processor, 8 GB RAM is used. In this work, we set the population size of all algorithms to 30, and each algorithm iterates 300 generations. BBMA is compared with the mayfly algorithm (MA), artificial electric field algorithm (AEFA) (Anita and Yadav, 2019), GA (Holland, 1992), PSO (Kennedy and Eberhart, 1995), imperialist competitive algorithm (ICA) (Atashpaz-Gargari and Lucas, 2008), seagull optimization algorithm (SOA) (Dhiman and Kumar, 2019), grasshopper optimization algorithm (GOA) (Storn and Price, 1997), grey wolf optimization (GWO) (Li et al., 2014), slime moth algorithm (SMA) (Saremi et al., 2017), differential evolution (DE) (Mirjalili et al., 2014), and animal migration optimization (AMO) (Li et al., 2020) in the best value, worst value, mean value, and standard deviation. In addition, in order to clearly prove the effectiveness of BBMA, the convergence curves, ANOVA test, fitness values for 30 runs, running time, and Wilcoxon rank-sum non-parametric statistical test (Derrac et al., 2011; Gibbons and Chakraborti, 2011) are also compared. Also, the minimum spanning tree is showed in spheres. The control parameters of each algorithm are as follows (Bi et al., 2021):

• BBMA: no parameters
• MA: positive attraction constants $\alpha_{1}=1,\alpha_{2}=1.5$ , visibility coefficient β=2 , nuptial dance coefficient d=0.1 , and random walk coefficient fl=0.1 (Zervoudakis and Tsafarakis, 2020)
• AEFA: Coulomb’s constant $K_{0}=500$ (Anita and Yadav, 2019)
• PSO: inertia weight g=0.2 , self-cognitive coefficient C1=0.7 , and social learning coefficient C2=1 (Kennedy and Eberhart, 1995)
• ICA: selection pressure is 1, assimilation coefficient is 2, revolution probability is 0.5, revolution rate is 0.1, and colony mean cost coefficient is 0.1 (Atashpaz-Gargari and Lucas, 2008)
• GA: crossover probability is 0.8, and mutation probability is 0.8 (Holland, 1992)
• GOA: intensity of attraction f=0.5 , attractive length scale l=1.5 , and the maximum and minimum values of the decline coefficient are $c_{\max}=1$ and $c_{\min}=0.00004$ (Dhiman and Kumar, 2019)
• GWO: convergence factor a decreases linearly from 2 to 0 (Storn and Price, 1997)
• SOA: $f_{c}$ that controls migration behavior decreases linearly from 2 to 0 (Li et al., 2014)
• SMA: foraging success probability z=0.03 (Saremi et al., 2017)
• DE: scaling factor F=0.5 , and crossover constant CR=0.5 (Mirjalili et al., 2014)
• AMO: no parameters (Li et al., 2020)

### Comparison of Algorithms in Low-Dimensional Cases

**FIGURE 3:**
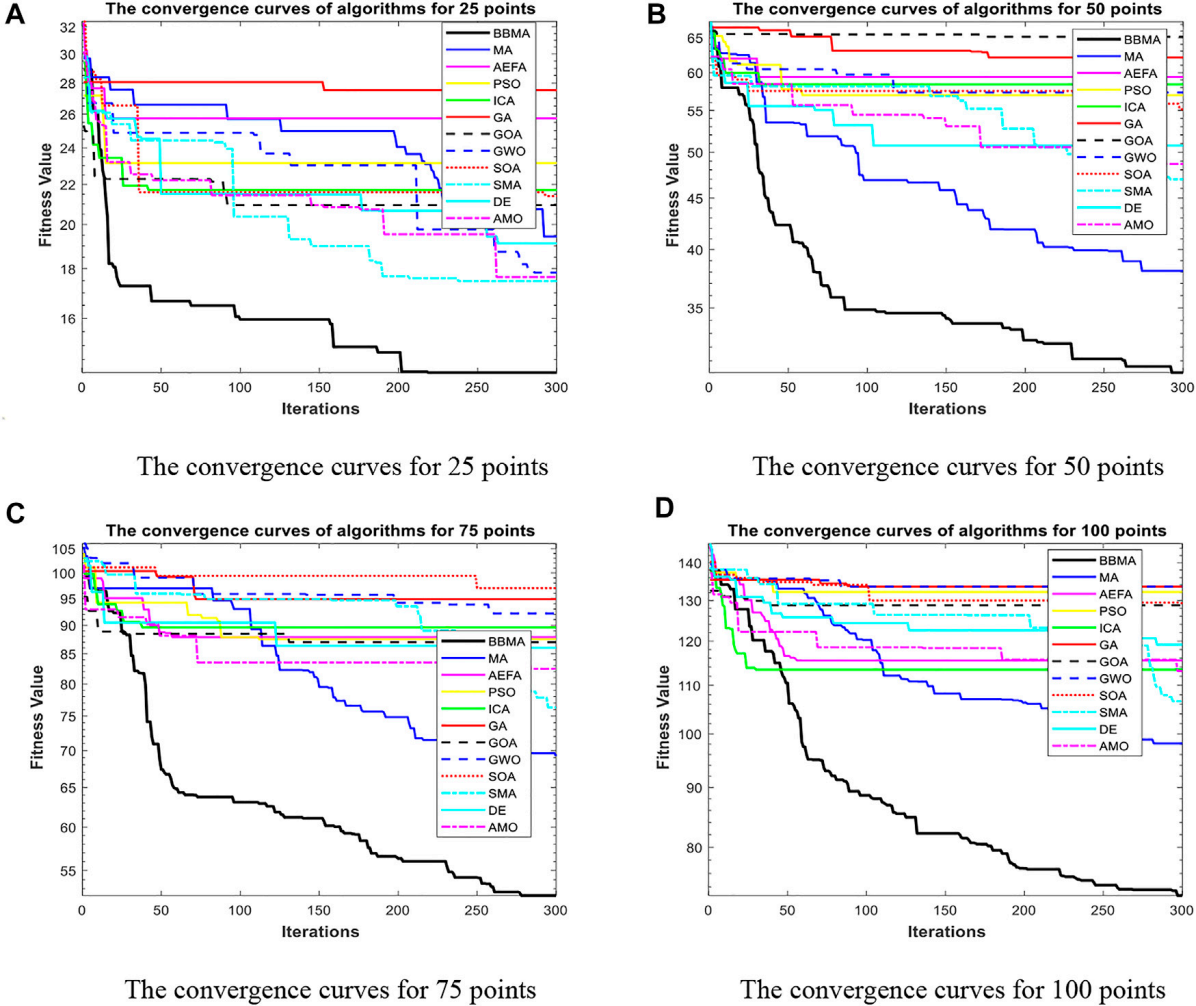
The convergence curves for low dimensions. **(A)** The convergence curves for 25 points. **(B)** The convergence curves for 50 points. **(C)**The convergence curves for 75 points. **(D)** The convergence curves for 100 points.

**FIGURE 4:**
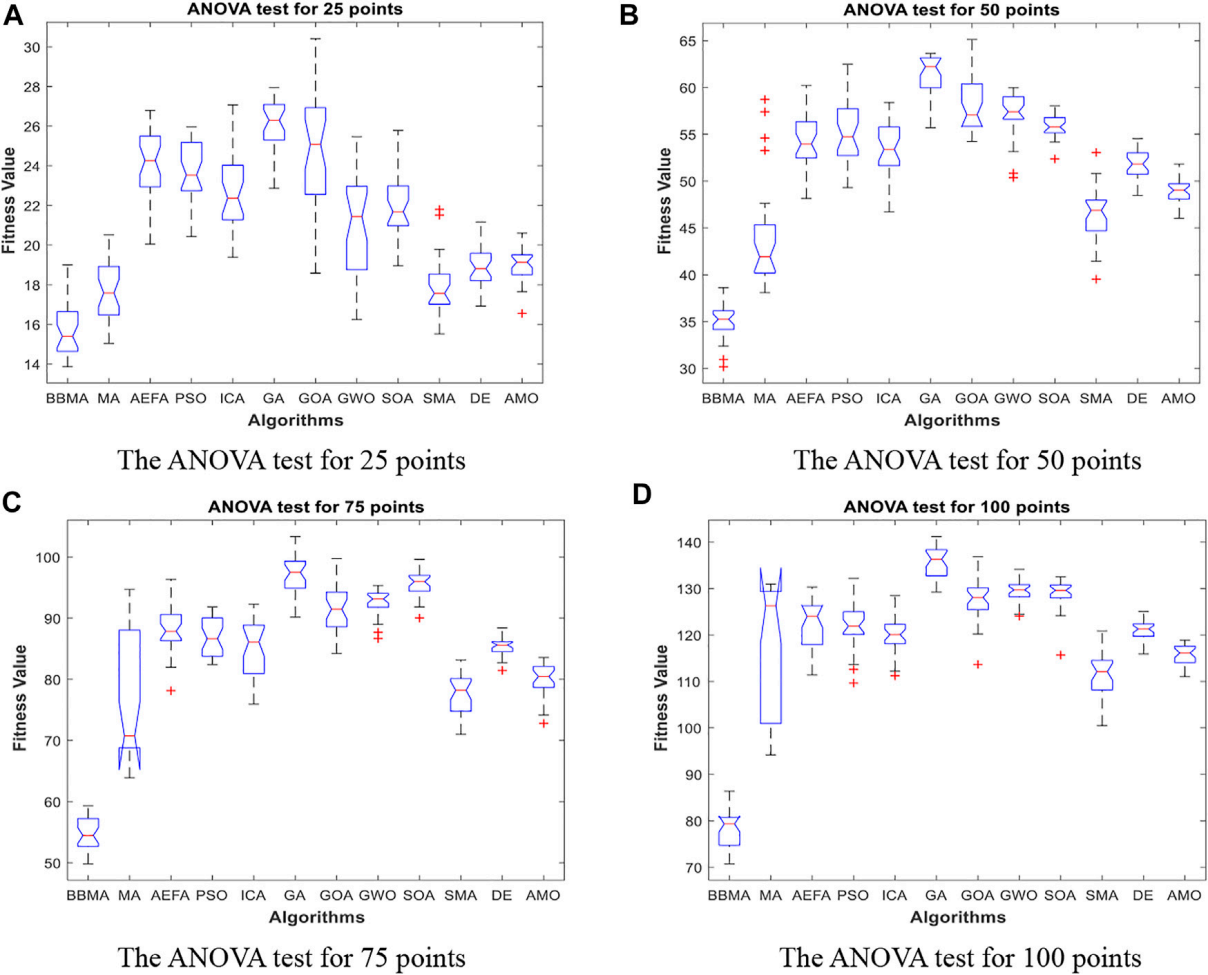
The ANOVA test for low dimensions. **(A)** The ANOVA test for 25 points. **(B)** The ANOVA test for 50 points. **(C)** The ANOVA test for 75 points. **(D)** The ANOVA test for 100 points.

**FIGURE 5:**
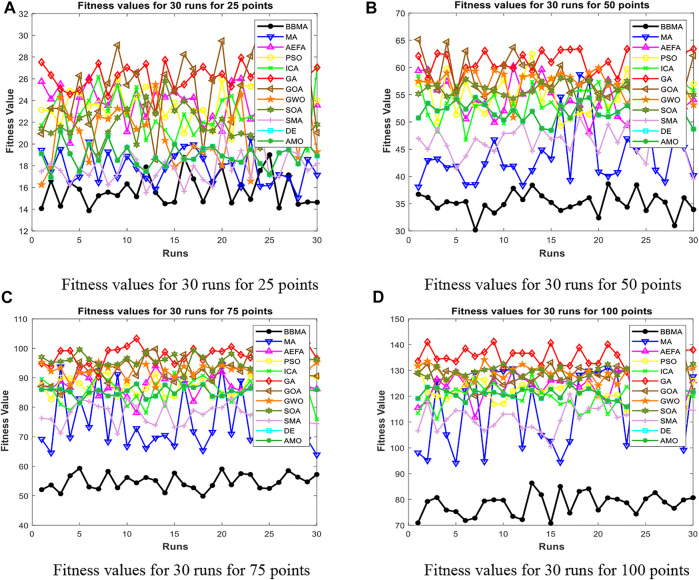
Fitness values for low dimensions. **(A)** Fitness values for 30 runs for 25 points. **(B)** Fitness values for 30 runs for 50 points. **(C)** Fitness values for 30 runs for 75 points. **(D)** Fitness values for 30 runs for 100 points.

Cases with 25, 50, 75, and 100 points are used to compare the performance of algorithms mentioned above, and the results of 30 runs are obtained. Table 1 gives the best value, worst value, mean value, standard deviation, and the ranking of mean value. The bold data indicate that it is the best value of the twelve algorithms. Figure 3 shows the convergence curves in these four situations, Figure 4 shows the ANOVA test results, Figure 5 shows the fitness values for 30 runs, and Figure 12A–D show the minimum spanning tree for four low-dimensional cases, where “ROOT” is the root of the minimum spanning tree. Figure 13A shows the average running time of 30 runs of 12 algorithms in four dimensions. Finally, the Wilcoxon rank-sum non-parametric test results in low-dimensional cases are shown in Table 2.

**TABLE 1:** Experimental results for the twelve algorithms for 25, 50, 75, and 100 points.

Points	Algorithms	Best	Worst	Mean	Std	Rank
25	BBMA	**13.6447**	**18.7544**	**15.8919**	1.0202	1
	MA	15.8860	20.9488	17.8316	1.2093	3
	AEFA, Bi et al. (2021)	19.3017	28.3577	23.9500	1.9339	11
	PSO, Bi et al. (2021)	18.6661	25.1622	22.1693	1.6687	8
	ICA, Bi et al. (2021)	18.1877	25.3421	21.8561	1.7114	6
	GA, Bi et al. (2021)	22.7281	28.0316	26.1953	1.2759	12
	GOA, Bi et al. (2021)	19.8519	26.1873	23.0678	1.6544	10
	GWO, Bi et al. (2021)	16.9782	27.0574	22.5108	2.3995	9
	SOA, Bi et al. (2021)	18.8431	24.4946	21.9361	1.3878	7
	SMA, Bi et al. (2021)	15.0231	19.8995	17.6528	1.2658	2
	DE	16.9290	21.1576	18.8842	1.0401	4
	AMO	16.5514	20.6010	19.0003	**0.8266**	5
50	BBMA	**28.4447**	**37.2789**	**34.8170**	1.7546	1
	MA	34.3380	58.6173	42.2226	4.4855	2
	AEFA, Bi et al. (2021)	50.2250	59.3597	55.1098	2.4704	8
	PSO, Bi et al. (2021)	44.8040	57.2689	51.5060	3.0896	5
	ICA, Bi et al. (2021)	45.7038	58.0749	52.4320	2.7355	7
	GA, Bi et al. (2021)	55.3518	64.8996	61.6177	2.3682	12
	GOA, Bi et al. (2021)	50.5135	58.4612	55.3181	2.0431	9
	GWO, Bi et al. (2021)	51.8792	59.9504	57.8262	1.5102	11
	SOA, Bi et al. (2021)	53.8901	58.4334	56.5395	**1.2611**	10
	SMA, Bi et al. (2021)	41.6473	54.8003	47.6913	3.0873	3
	DE	48.4642	54.5281	51.7636	1.6024	6
	AMO	45.0359	51.8286	48.9038	1.4479	4
75	BBMA	**48.6839**	**59.3288**	**54.7791**	3.5118	1
	MA	59.7131	93.1400	76.1626	11.7156	2
	AEFA, Bi et al. (2021)	78.5702	93.0950	87.1792	3.7266	8
	PSO, Bi et al. (2021)	76.2217	83.4455	83.4455	3.0984	5
	ICA, Bi et al. (2021)	74.9482	95.2576	85.2616	4.4489	6
	GA, Bi et al. (2021)	92.5715	104.4691	98.6174	2.8997	12
	GOA, Bi et al. (2021)	82.9354	95.4337	87.8587	3.1684	9
	GWO, Bi et al. (2021)	89.7958	96.8943	93.2198	1.9073	10
	SOA, Bi et al. (2021)	88.1649	99.3988	95.7273	2.6763	11
	SMA, Bi et al. (2021)	73.2677	88.9004	82.6417	4.0689	4
	DE	81.4543	88.4035	85.4406	**1.5659**	7
	AMO	72.7859	83.5528	79.9430	2.6396	3
100	BBMA	**69.2455**	**82.8067**	**76.9687**	3.358	1
	MA	93.0942	132.0213	117.2067	13.3847	5
	AEFA, Bi et al. (2021)	108.6233	132.4447	121.9061	5.5434	8
	PSO, Bi et al. (2021)	105.3880	122.9785	114.8626	4.8750	2
	ICA, Bi et al. (2021)	113.1387	130.8257	120.4606	4.4412	6
	GA, Bi et al. (2021)	122.6827	141.3354	136.5707	3.5123	12
	GOA, Bi et al. (2021)	115.5154	131.2544	124.3536	4.3611	9
	GWO, Bi et al. (2021)	115.5757	132.9414	128.5117	3.6147	10
	SOA, Bi et al. (2021)	124.2706	132.8262	129.5164	2.4841	11
	SMA, Bi et al. (2021)	102.8094	127.4766	116.7717	4.8956	4
	DE	115.9142	125.0204	120.9535	**2.0062**	7
	AMO	111.0088	118.8614	115.6808	2.3881	3

The optimal values are shown in bold.

**TABLE 2:** Wilcoxon rank-sum test results in low dimensions.

Points	MA	AEFA	PSO	ICA	GA	GOA	GWO	SOA	SMA	DE	AMO
25	1.0570E-04	1.7344E-06	1.9209E-06	1.7344E-06	1.7344E-06	1.7344E-06	2.1266E-06	1.7344E-06	1.4773E-04	5.7517e-06	1.7344E-06
50	1.7344E-06	1.7344E-06	1.7344E-06	1.9209E-06	1.7344E-06	1.7344E-06	1.7344E-06	1.7344E-06	1.7344E-06	1.7344E-06	1.7344E-06
75	1.7344E-06	1.7344E-06	1.7344E-06	1.7344E-06	1.7344E-06	1.7344E-06	1.7344E-06	1.7344E-06	1.7344E-06	1.7344E-06	1.7344E-06
100	1.7344E-06	1.7344E-06	1.7344E-06	1.7344E-06	1.7344E-06	1.7344E-06	1.7344E-06	1.7344E-06	1.7344E-06	1.7344E-06	1.7344E-06

The comparison results for 25 points are shown in Table 1. BBMA performs best in the best, worst, and mean value, but its standard deviation is 1.0202 which is worse than that of AMO, while the algorithm with the worst performance is GA. Figure 3A is the convergence curves of all algorithms; obviously, BBMA has the fastest convergence speed of twelve mentioned algorithms. As can be seen from Figure 4A, BBMA has the highest stability, and GWO is the worst. Figure 5A shows that, among the fitness values of 30 runs, BBMA is better than other algorithms in most cases, but AMO is three times better than BBMA, SMA is three times better than it, and MA is six times better than it. Figure 12A shows the minimum spanning tree for 25 points.

The comparison results of twelve algorithms at 50 points are shown in Table 1. It can be seen that the best value, worst value, and mean value of BBMA are the best, but the standard deviation ranks fifth, behind SOA, AMO, GWO, and DE. Figure 3B and Figure 4B show the convergence curve and analysis of variance results, respectively. By observing the convergence curve, we can clearly see that BBMA has the highest accuracy and the fastest convergence speed. Also, the result of variance analysis shows that BBMA is stable for solving this problem. The fitness values for 30 runs is shown in Figure 5B, and it can be seen that BBMA outperforms all other algorithms in 30 runs. Figure 12B shows the minimum spanning tree for 50 points.

The comparison of 75 points is shown in Table 1. BBMA is better than others in the best value, worst value, and mean value, but its standard deviation is 3.5118 which is worse than the standard deviation of PSO, GA, GOA, GWO, and SOA. Besides, GA has the worst accuracy. By observing Figure 3C, it can be noticed that both convergence accuracy and convergence speed are the highest for BBMA. As can be seen from Figure 4C, BBMA is still stable. The fitness values of 30 runs and the minimum spanning tree of 75 points can be seen in Figure 5C and Figure 12C.

The experience results for 100 points are shown in Table 1. The performance of BBMA is superior to that of others in the best, worst, and mean value. The best value of BBMA is 69.2455, and the best value of MA is 93.0942. BBMA is 25.83% better than the original algorithm. Figure 3D shows that BBMA has the highest accuracy and it still has excellent exploitation ability when other algorithms are stuck in a local optimal value. Figure 4D shows that BBMA has high stability. Figure 5D and Figure 12D show the fitness values for 30 runs and the minimum spanning tree path optimized for 100 points.

It can be seen from Figure 13A that BBMA runs the longest at each case, and GA and GWO are the two fastest algorithms. In addition to the running time, by comparing with other eleven algorithms, BBMA has the best performance in low dimensions. In addition, this study statistically tests the proposed algorithm. The Wilcoxon rank-sum non-parametric test results are shown in Table 2. BBMA is tested with others at the d=0.05 significance level. If *p* values in the table are all less than 0.05, it will prove that BBMA is obviously better than others. Statistically, the experimental results are significant.

### Comparison of Algorithms for Medium-Dimensional Cases

**FIGURE 6:**
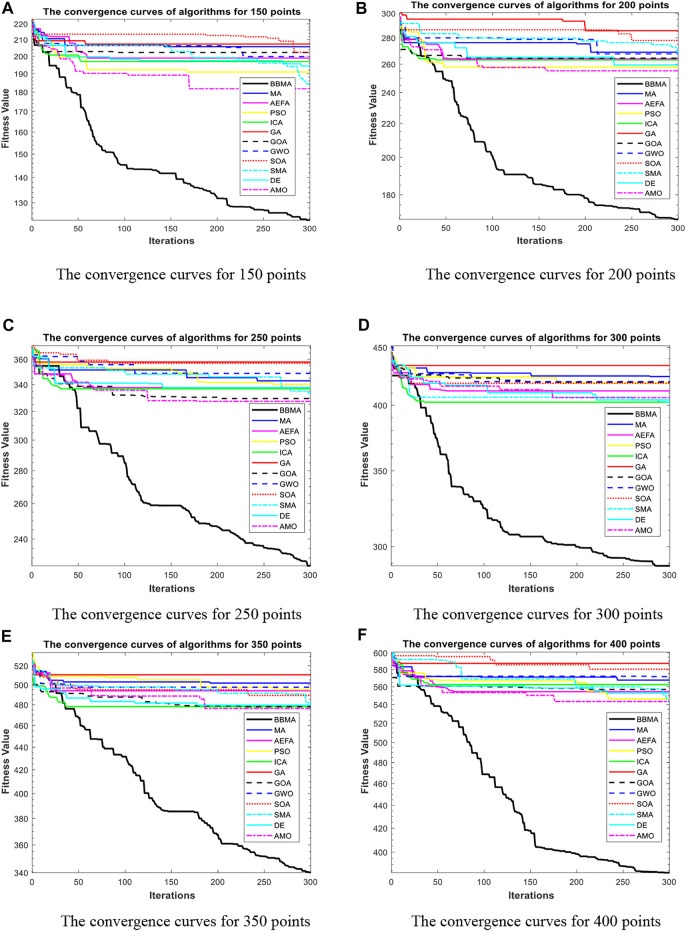
The convergence curves for medium dimensions. **(A)** The convergence curves for 150 points. **(B)** The convergence curves for 200 points. **(C)** The convergence curves for 250 points. **(D)** The convergence curves for 300 points. **(E)** The convergence curves for 350 points. **(F)** The convergence curves for 400 points.

**FIGURE 7:**
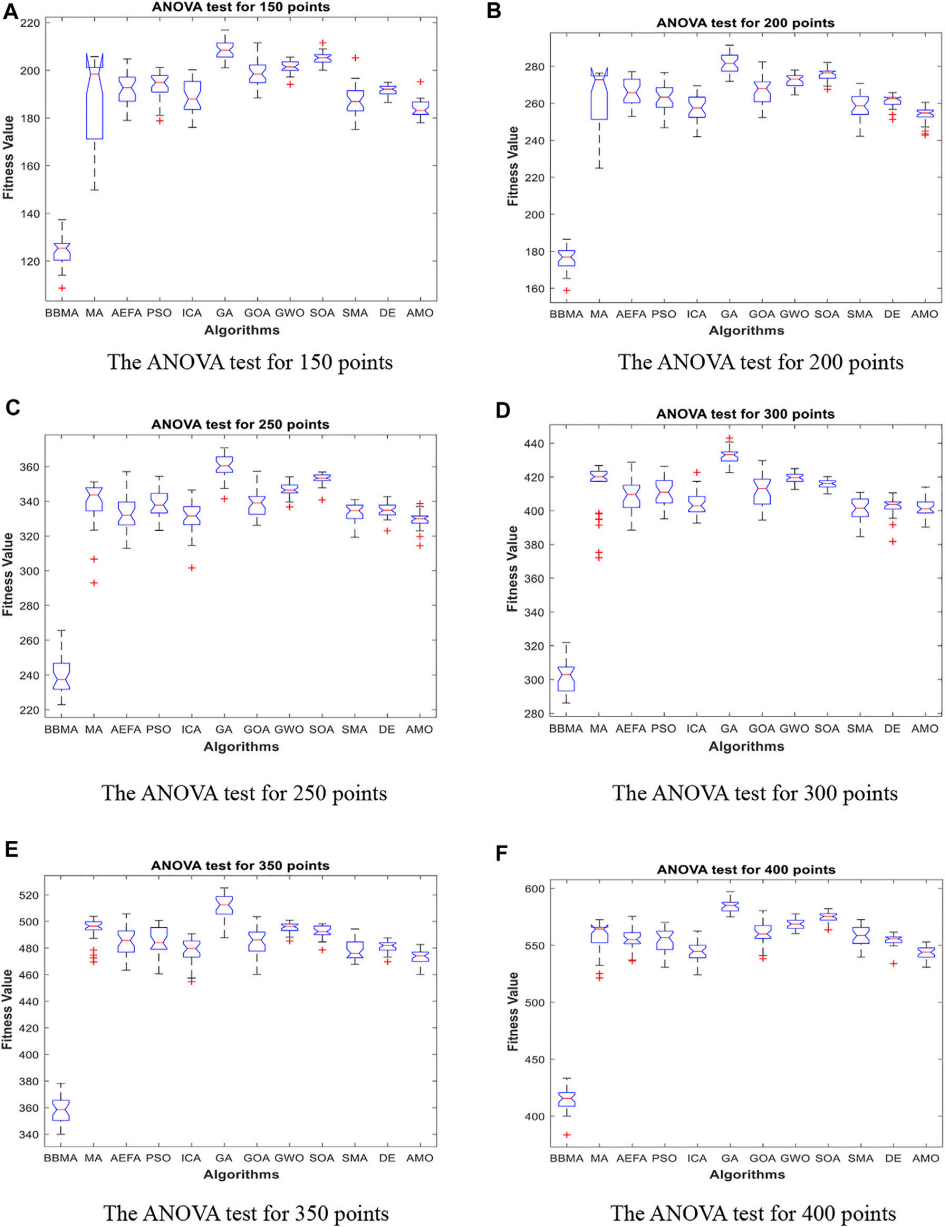
The ANOVA test for medium dimensions. **(A)** The ANOVA test for 150 points. **(B)** The ANOVA test for 200 points. **(C)** The ANOVA test for 250 points. **(D)** The ANOVA test for 300 points. **(E)** The ANOVA test for 350 points. **(F)** The ANOVA test for 400 points.

**FIGURE 8:**
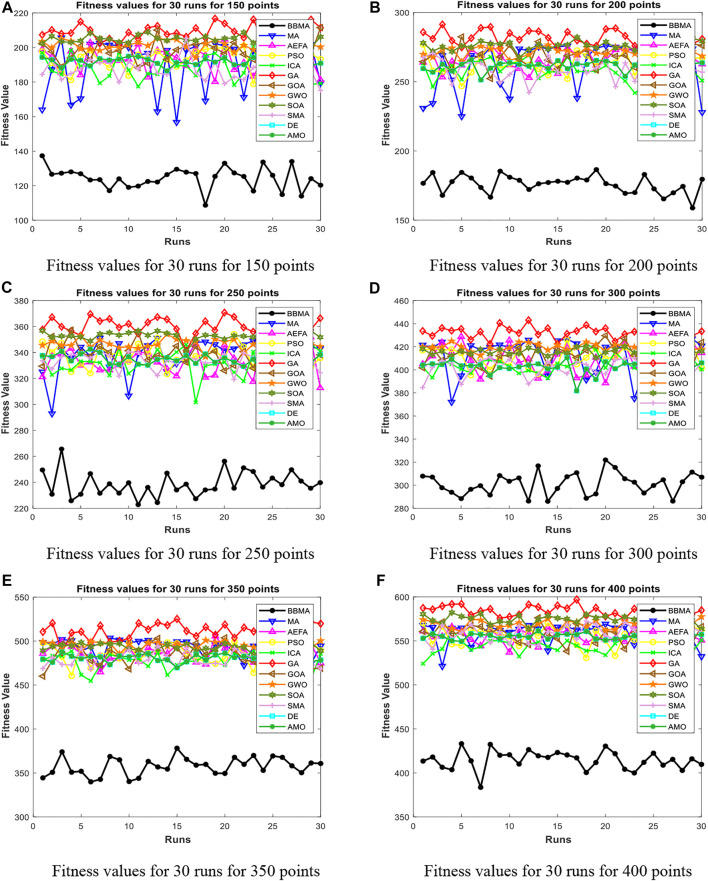
Fitness values for medium dimensions. **(A)** Fitness values for 30 runs for 150 points. **(B)** Fitness values for 30 runs for 200 points. **(C)** Fitness values for 30 runs for 250 points. **(D)** Fitness values for 30 runs for 300 points. **(E)** Fitness values for 30 runs for 350 points. **(F)** Fitness values for 30 runs for 400 points.

In this section, BBMA and other eleven algorithms are tested in six medium-dimensional cases from 150 points, 200 points, 250 points, 300 points, 350 points, and 400 points. Table 3 records the best, worst, and mean value, standard deviation, and the ranking of the mean value. The bold data indicates that it is the best value of the twelve algorithms. Figure 6 shows the convergence curves of these six cases, and Figure 7 shows the ANOVA test results for each case. The fitness values for 30 runs are shown in Figure 8. Also, the minimum spanning tree for these cases is listed in Figure 12E–J, where “ROOT” is the root of the minimum spanning tree. In addition, Figure 13B shows the average running time of the twelve algorithms in different dimensions. Finally, the Wilcoxon rank-sum non-parametric test results in medium-dimensional cases are shown in Table 4.

**TABLE 3:** Experimental results for the twelve algorithms for 150, 200, 250, 300, 350, and 400 points.

Points	Algorithms	Best	Worst	Mean	Std	Rank
150	BBMA	**108.7044**	**128.5096**	**121.2003**	6.0033	1
	MA	159.5308	203.8459	187.3684	15.6695	5
	AEFA, Bi et al. (2021)	177.1469	195.8829	186.8330	5.2970	4
	PSO, Bi et al. (2021)	171.8660	192.5427	183.8670	5.7697	2
	ICA, Bi et al. (2021)	170.6246	195.5246	187.3846	5.6297	6
	GA, Bi et al. (2021)	203.0229	219.0789	210.6552	4.6014	12
	GOA, Bi et al. (2021)	181.2130	205.7132	193.7471	5.9105	9
	GWO, Bi et al. (2021)	198.1928	206.1991	201.7777	**2.0473**	10
	SOA, Bi et al. (2021)	199.3280	210.1148	205.7050	2.7155	11
	SMA, Bi et al. (2021)	184.0786	201.3937	193.6468	4.2731	8
	DE	186.4958	194.9518	191.7888	2.0882	7
	AMO	178.0064	195.1718	183.9280	3.8121	3
200	BBMA	**158.8478**	**186.5355**	**175.9532**	6.4658	1
	MA	223.8146	277.646	255.0136	20.2298	4
	AEFA, Bi et al. (2021)	250.5322	284.2144	263.1465	7.7213	8
	PSO, Bi et al. (2021)	241.6883	269.7025	254.6991	6.8041	3
	ICA, Bi et al. (2021)	243.7737	273.2664	257.9959	7.6342	5
	GA, Bi et al. (2021)	268.7868	296.2953	284.4061	5.8340	12
	GOA, Bi et al. (2021)	254.5294	271.1409	264.3730	4.4879	9
	GWO, Bi et al. (2021)	266.3731	277.0690	273.0821	2.7613	10
	SOA, Bi et al. (2021)	273.1784	282.3387	277.8522	**2.4687**	11
	SMA, Bi et al. (2021)	241.7519	275.1255	260.9995	8.6126	6
	DE	251.2678	265.8108	261.2612	3.3148	7
	AMO	242.7951	260.4549	253.8667	4.4861	2
250	BBMA	**222.9245**	**254.7082**	**235.7813**	8.9879	1
	MA	312.5237	352.2358	342.2936	10.0794	9
	AEFA, Bi et al. (2021)	316.4191	357.8107	336.8645	8.4107	5
	PSO, Bi et al. (2021)	314.2942	354.3313	337.7788	7.7460	6
	ICA, Bi et al. (2021)	321.5577	348.8649	332.0370	6.6376	3
	GA, Bi et al. (2021)	352.2579	372.6212	361.2289	6.5794	12
	GOA, Bi et al. (2021)	320.2223	349.6514	338.6692	7.0944	7
	GWO, Bi et al. (2021)	337.3628	353.7242	346.8830	**4.0648**	10
	SOA, Bi et al. (2021)	339.6638	358.2409	351.6310	4.4290	11
	SMA, Bi et al. (2021)	319.2010	348.9050	339.7043	6.3158	8
	DE	322.9296	342.6385	334.8455	4.1294	4
	AMO	314.4014	338.6204	329.3998	5.2065	2
300	BBMA	**275.7485**	**320.7405**	**298.7668**	10.9117	1
	MA	379.1592	425.7837	413.7186	13.8915	9
	AEFA, Bi et al. (2021)	381.9766	432.1147	406.6118	10.8638	6
	PSO, Bi et al. (2021)	390.3274	425.3140	412.8644	8.6242	8
	ICA, Bi et al. (2021)	383.9086	415.8944	400.3720	8.5896	2
	GA, Bi et al. (2021)	422.2974	448.4215	437.9276	5.7820	12
	GOA, Bi et al. (2021)	388.2968	421.3079	408.2574	8.2339	7
	GWO, Bi et al. (2021)	416.0361	425.4912	420.2078	**2.5851**	11
	SOA, Bi et al. (2021)	404.6652	424.1153	414.6620	4.0100	10
	SMA, Bi et al. (2021)	391.2315	417.1096	404.5279	6.1692	5
	DE	381.7567	410.6107	402.4801	5.3663	4
	AMO	390.2933	413.9668	401.9473	5.3906	3
350	BBMA	**330.6316**	**379.8724**	**356.8802**	11.8212	1
	MA	452.2005	501.0686	488.1878	14.4075	9
	AEFA, Bi et al. (2021)	461.6761	501.5019	483.2021	9.3857	6
	PSO, Bi et al. (2021)	453.2908	498.2213	484.1515	10.7357	8
	ICA, Bi et al. (2021)	456.7770	493.9439	475.2845	9.5585	3
	GA, Bi et al. (2021)	494.1726	526.2063	514.2878	8.1990	12
	GOA, Bi et al. (2021)	461.8404	494.6949	479.9414	8.0381	4
	GWO, Bi et al. (2021)	485.3296	502.2586	495.3422	**3.6271**	11
	SOA, Bi et al. (2021)	469.5365	498.2843	490.9486	6.2352	10
	SMA	466.6158	494.4263	483.9335	7.5615	7
	DE	469.6890	487.5686	480.7679	4.0358	5
	AMO	460.0170	482.6614	473.4664	5.3265	2
400	BBMA	**382.1640**	**438.9316**	**415.9261**	13.1962	1
	MA	508.5346	569.2624	551.9432	18.5577	4
	AEFA, Bi et al. (2021)	533.7028	575.2738	558.5251	10.7157	8
	PSO, Bi et al. (2021)	535.9726	563.4369	552.9052	7.1746	5
	ICA, Bi et al. (2021)	527.6904	576.9956	549.9101	11.2322	3
	GA, Bi et al. (2021)	562.2246	601.2813	586.7195	7.9634	12
	GOA, Bi et al. (2021)	533.5131	573.5504	555.9482	10.8102	7
	GWO, Bi et al. (2021)	558.2885	576.4934	568.7354	**4.3849**	10
	SOA, Bi et al. (2021)	562.1377	580.3801	575.1273	4.3925	11
	SMA, Bi et al. (2021)	545.4407	577.0593	561.7902	8.3622	9
	DE	534.0305	561.5491	554.6098	4.9569	6
	AMO	530.8524	552.8364	543.4986	5.3476	2

The optimal values are shown in bold.

**TABLE 4:** Wilcoxon rank-sum test results in medium dimensions.

Points	MA	AEFA	PSO	ICA	GA	GOA	GWO	SOA	SMA	DE	AMO
150	1.7344E-06	1.7344E-06	1.7344E-06	1.7344E-06	1.7344E-06	1.7344E-06	1.7344E-06	1.7344E-06	1.7344E-06	1.7344E-06	1.7344E-06
200	1.7344E-06	1.7344E-06	1.7344E-06	1.7344E-06	1.7344E-06	1.7344E-06	1.7344E-06	1.7344E-06	1.7344E-06	1.7344E-06	1.7344E-06
250	1.7344E-06	1.7344E-06	1.7344E-06	1.7344E-06	1.7344E-06	1.7344E-06	1.7344E-06	1.7344E-06	1.7344E-06	1.7344E-06	1.7344E-06
300	1.7344E-06	1.7344E-06	1.7344E-06	1.7344E-06	1.7344E-06	1.7344E-06	1.7344E-06	1.7344E-06	1.7344E-06	1.7344E-06	1.7344E-06
350	1.7344E-06	1.7344E-06	1.7344E-06	1.7344E-06	1.7344E-06	1.7344E-06	1.7344E-06	1.7344E-06	1.7344E-06	1.7344E-06	1.7344E-06
400	1.7344E-06	1.7344E-06	1.7344E-06	1.7344E-06	1.7344E-06	1.7344E-06	1.7344E-06	1.7344E-06	1.7344E-06	1.7344E-06	1.7344E-06

Table 3 displays the experience results of 150 points and 200 points. Also, the statistical data shown in these tables reflect the great difference between different algorithms in searching ability. We can discover that except standard deviation, the best value, worst value, and mean value of BBMA are all the optimal. Also, the performance of GA is the worst. Figures 6A,B are the convergence curves for the two cases, and it can be seen that BBMA has a faster convergence speed and accuracy and strong exploration ability. Figures 7A,B show the analysis of variance results for the two cases, and we can see that the stability of BBMA is at a relatively high level. Figures 8A,B are the fitness values in 30 runs for 150 and 200 points. The MST for the two cases can be found in Figures 12E,F.

Table 3 also shows the comparison results of different algorithms at 250 points and 300 points. BBMA is the best in the best, worst, and mean value, and GA is the worst. However, as for the standard deviation, GWO is the best at 250 and 300 points. Figures 6C,D show the convergence curves in these two cases. The convergence speed and accuracy of BBMA are much superior to others. When other algorithms fall into local optimization, it still has good performance. The results of analysis of variance can be seen in Figures 7C,D, and BBMA has high stability. Figures 8C,D show the curves of the fitness values of 12 algorithms running independently for 30 times in these two cases. The search accuracy of BBMA is much better than that of the other 11 algorithms. Figures 12G,H show the MST at 250 points and 300 points, respectively.

The situation at 350 points and 400 points is shown in Table 3. BBMA performs best in the best, worst, and mean value. Compared with the best value of MA, the accuracy of BBMA is improved by 26.88% at 350 points and 24.85% at 400 points. As shown in Figures 6E,F, with the growth of dimension, the performance of BBMA is getting better and better. Most algorithms fall into local optimal solution at generation 100, but BBMA always has strong search ability. Figures 7E,F show the analysis of variance results in two cases, Figures 8E,F show the fitness values of 30 runs, and Figures 12I,F show the MST. It can be seen that BBMA has high stability and has better ability to solve spherical MST problems in medium-dimension cases than in low-dimension cases.

In addition, Figure 13B shows that the average running time of BBMA is the longest in the six cases. Compared with other algorithms, MA, DE, and AMO also run longer. Through the abovementioned analysis, we have noticed that BBMA has the outstanding performance in the medium-dimensional cases. The Wilcoxon rank-sum test results are shown in Table 4. Similarly, the *p* values in the table are all less than 0.05, which proves that BBMA algorithm is better than others in medium dimensions.

### Comparison of Algorithms for High-Dimensional Cases

In *Comparison of Algorithms in Low-Dimensional Cases* and *Comparison of Algorithms for Medium-Dimensional Cases*, BBMA has been compared with other 11 algorithms in low and medium dimensions. BBMA shows very superior performance. Most of the problems encountered in real life are complex and high-dimensional problems, so in this section, BBMA and MA are tested in higher dimensions where *n* = 500, 600, 700, 800, 900, and 1,000 (see Table 5).

**TABLE 5:** Experimental results for the two algorithms for 500, 600, 700, 800, 900, and 1,000 points.

Points	Algorithms	Best	Worst	Mean	Std	Rank
500	BBMA	**526.7288**	**576.1991**	**551.4301**	**10.9296**	1
	MA	671.1059	726.2274	713.1005	13.0341	2
600	BBMA	**661.5117**	**720.9847**	**687.1937**	15.486	1
	MA	827.6303	881.1784	869.5585	**11.0172**	2
700	BBMA	**795.8841**	**887.1201**	**826.7429**	20.7736	1
	MA	973.2014	1029.001	1014.8361	**14.729**	2
800	BBMA	**920.5137**	**1003.6668**	**961.2036**	21.599	1
	MA	1150.4318	1184.9458	1172.4426	**9.8713**	2
900	BBMA	**1072.6695**	**1167.2939**	**1107.1993**	28.7825	1
	MA	1285.7222	1342.1227	1324.583	**11.7061**	2
1000	BBMA	**1167.5382**	**1344.4605**	**1248.6469**	34.2288	1
	MA	1427.1684	1490.0547	1476.1061	**12.4662**	2

The optimal values are shown in bold.

**FIGURE 9:**
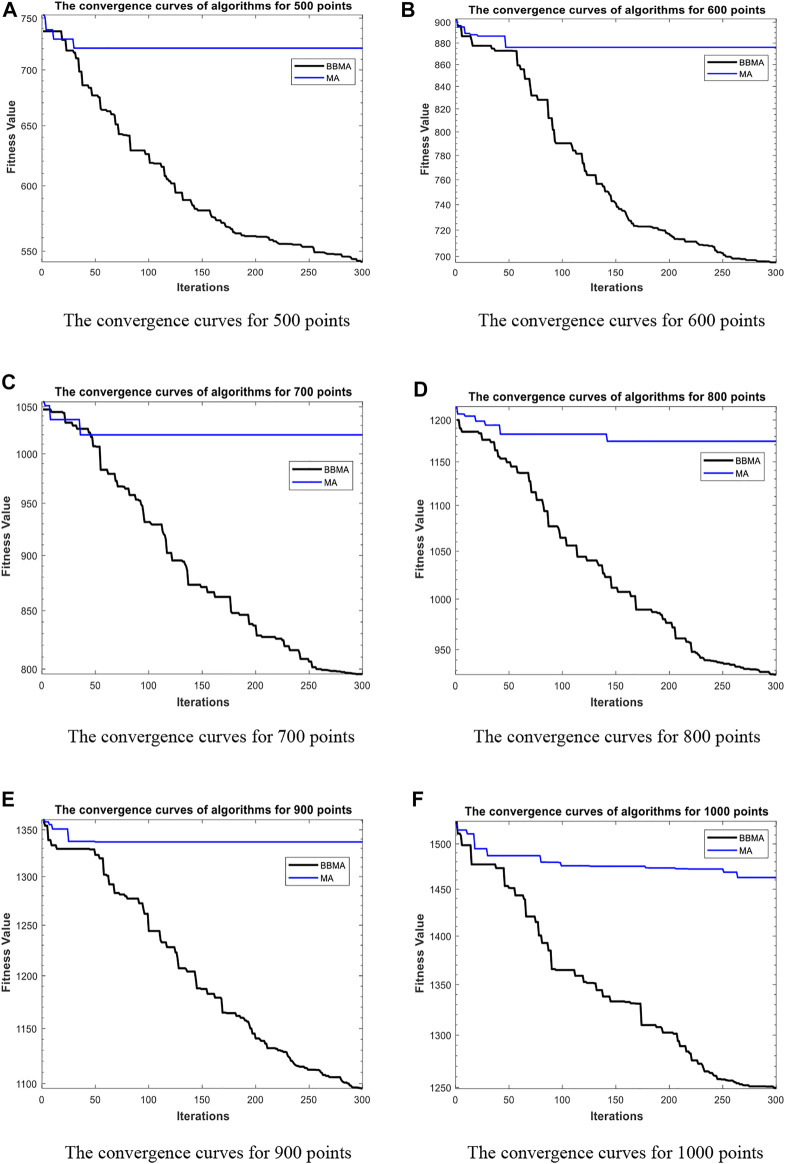
The convergence curves for high dimensions. **(A)** The convergence curves for 500 points. **(B)** The convergence curves for 600 points. **(C)** The convergence curves for 700 points. **(D)** The convergence curves for 800 points. **(E)** The convergence curves for 900 points. **(F)** The convergence curves for 1000 points.

**FIGURE 10:**
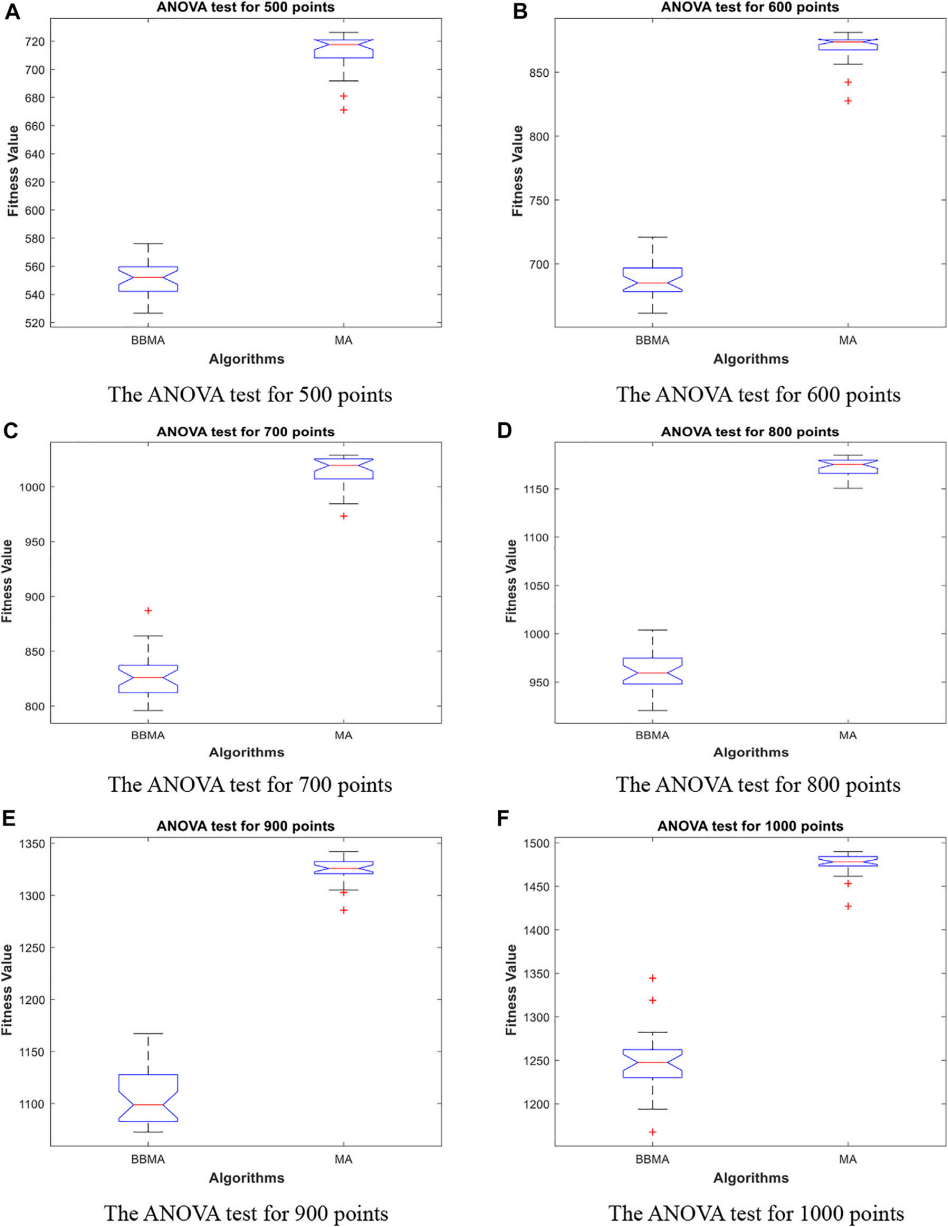
The ANOVA test for high dimensions. **(A)** The ANOVA test for 500 points. **(B)** The ANOVA test for 600 points. **(C)** The ANOVA test for 700 points. **(D)** The ANOVA test for 800 points. **(E)** The ANOVA test for 900 points. **(F)** The ANOVA test for 1000 points.

**FIGURE 11:**
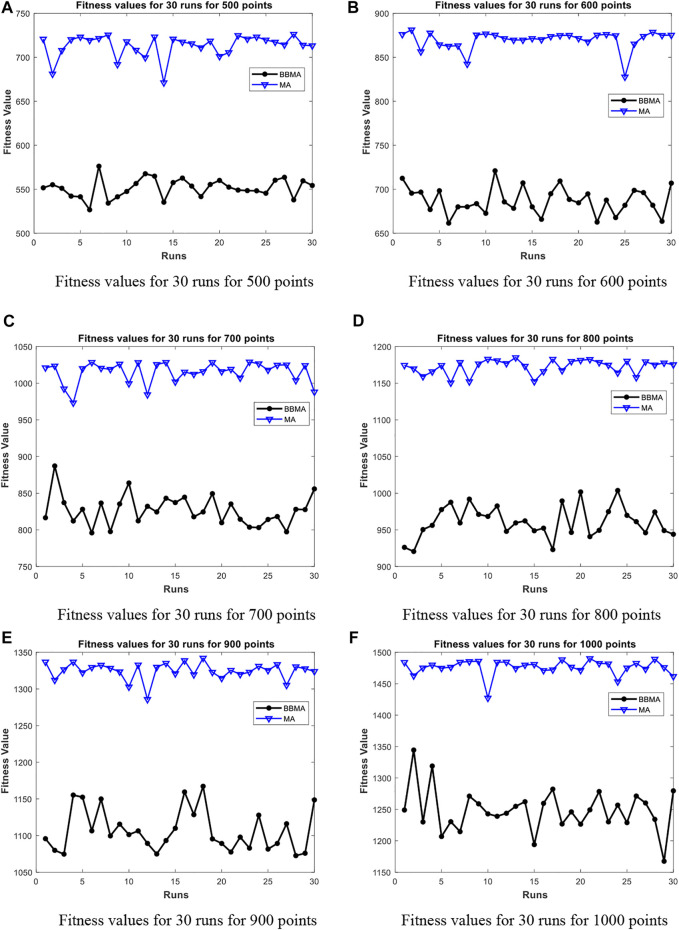
Fitness values for high dimensions. **(A)** Fitness values for 30 runs for 500 points. **(B)** Fitness values for 30 runs for 600 points. **(C)** Fitness values for 30 runs for 700 points. **(D)** Fitness values for 30 runs for 800 points. **(E)** Fitness values for 30 runs for 900 points. **(F)** Fitness values for 30 runs for 1000 points.

**FIGURE 12:**
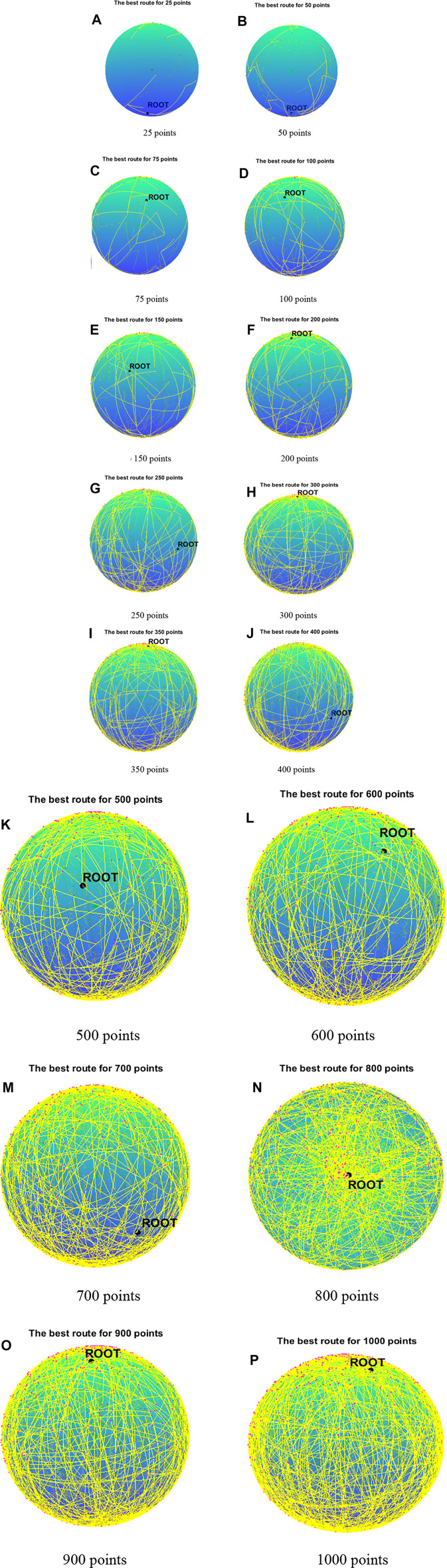
The minimum spanning tree for 16 cases. **(A)** 25 points. **(B)** 50 points. **(C)** 75 points. **(D)** 100 points. **(E)** 150 points. **(F)** 200 points. **(G)** 250 points. **(H)** 300 points. **(G)** 250 points. **(H)** 300 points. **(I)** 350 points. **(J)** 400 points. **(K)** 500 points. **(L)** 600 points. **(M)** 700 points. **(N)** 800 points. **(O)** 900 points. **(P)** 1000 points.

**FIGURE 13:**
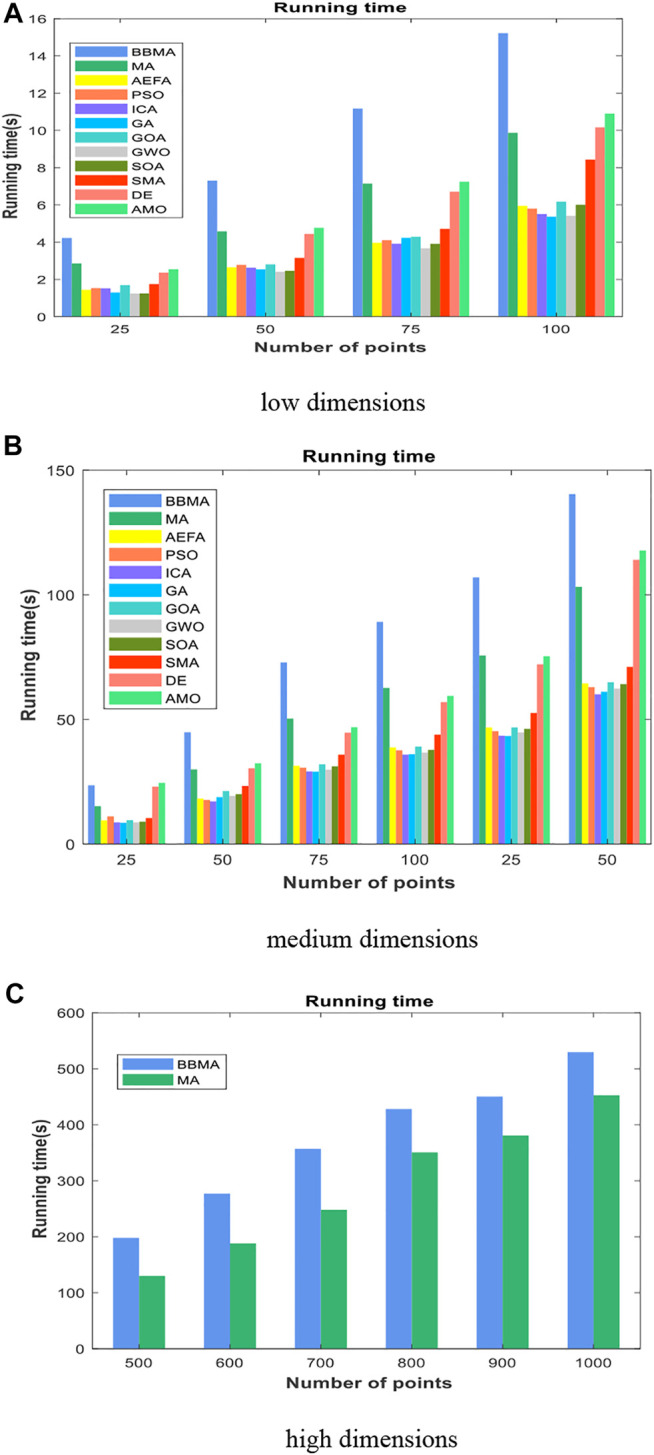
The average running time for 16 cases. **(A)** Low dimensions. **(B)** Medium dimensions. **(C)** High dimensions.

Table 5 shows the comparison results of BBMA and MA in six high-dimensional cases and also compares the best, worst, and mean value and standard deviation. The bold data indicate that it is the optimal result of the two. It can be seen that BBMA is superior to its original algorithm in the first three items in each case. The convergence curves of the two algorithms are shown in Figures 9A–F; obviously, the convergence speed and convergence accuracy of BBMA are better, and both exploration and exploitation capabilities have been greatly improved. The results of analysis of variance are shown in Figures 10A–F, and MA is more stable than BBMA. The fitness values of 30 independent operations are shown in Figures 11A–F. Also, Figures 12A–P show the MST of BBMA in different cases, where “ROOT” is the root of the MST, and the minimum spanning tree produced by BBMA is of high quality. Figure 13C is a histogram of the average running time of BBMA and MA, and we find that BBMA runs longer. Finally, Table 6 shows the results of the Wilcoxon rank-sum test in high-dimensional cases. The *p* values are so small that we can know that BBMA is significantly better than MA.

**TABLE 6:** Wilcoxon rank-sum test results in high dimensions.

Points	500	600	700	800	900	1000
MA	1.7344E-06	1.7344E-06	1.7344E-06	1.7344E-06	1.7344E-06	1.7344E-06

## Conclusion and Future Work

Mayfly algorithm is a new population-based bioinspired algorithm, which has strong ability to solve continuous problems. It combines the advantages of PSO, FA, and GA and has superior exploration ability, high solution accuracy, and fast convergence. It improves the shortcoming that MA has many initial parameters and the parameters have a large impact on the results. Furthermore, Lévy flight is used for updating the position of the optimal male and excellent female to help the algorithm escape from local optimal solution. In addition, in order to make effective use of the search space, a cross-border punishment mechanism similar to “mirror wall” is used to deal with cross-border individuals. In order to demonstrate the effectiveness of BBMA, the MST problems are solved on a sphere. Compared with MA, AEFA, GA, PSO, ICA, SOA, GOA, GWO, SMA, DE, and AMO in 16 different cases, the test results show that BBMA has superior solving ability, and the higher the dimension is, the more obvious the superiority of BBMA will be. Therefore, BBMA is a good method for large-scale problems in real life. According to the NFL theorem, there is no algorithm that has superior performance for any problem. BBMA has some limitations in solving the spherical MST problems: its running time is relatively long, and its stability needs to be improved. In the future, BBMA will be further applied for solving the spherical MST problems in real life, such as removing noise on the femoral surface and directional location estimators (Kirschstein et al., 2013; Kirschstein et al., 2019). Also, it will be applied to other practical applications, such as logistics center location, path planning, weather forecast, and charging station address selection.

## Data Availability

The original contributions presented in the study are included in the article, further inquiries can be directed to the corresponding authors.
